# Entropy-Based Concentration and Instantaneous Frequency of TFDs from Cohen’s, Affine, and Reassigned Classes

**DOI:** 10.3390/s22103727

**Published:** 2022-05-13

**Authors:** David Bačnar, Nicoletta Saulig, Irena Petrijevčanin Vuksanović, Jonatan Lerga

**Affiliations:** 1 Department of Computer Engineering, Faculty of Engineering, University of Rijeka, Vukovarska 58, 51000 Rijeka, Croatia; dbacnar@riteh.hr; 2 Department of Engineering, Juraj Dobrila University of Pula, Zagrebačka 30, 52100 Pula, Croatia; nsaulig@unipu.hr; 3 Ministry of the Interior, 10000 Zagreb, Croatia; irenapv@uniri.hr; 4 Center for Artificial Intelligence and Cybersecurity, University of Rijeka, Radmile Matejčić 2, 51000 Rijeka, Croatia

**Keywords:** time–frequency representation, Cohen’s class, affine class, reassigned class, time–frequency resolution, time–frequency concentration, Rényi entropy, instantaneous frequency

## Abstract

This paper explores three groups of time–frequency distributions: the Cohen’s, affine, and reassigned classes of time–frequency representations (TFRs). This study provides detailed insight into the theory behind the selected TFRs belonging to these classes. Extensive numerical simulations were performed with examples that illustrate the behavior of the analyzed TFR classes in the joint time–frequency domain. The methods were applied both on synthetic and real-life non-stationary signals. The obtained results were assessed with respect to time–frequency concentration (measured by the Rényi entropy), instantaneous frequency (IF) estimation accuracy, cross-term presence in the TFRs, and the computational cost of the TFRs. This study gives valuable insight into the advantages and limitations of the analyzed TFRs and assists in selecting the proper distribution when analyzing given non-stationary signals in the time–frequency domain.

## 1. Introduction

### 1.1. Time–Frequency Analysis

The most common way to represent a signal is as a time-dependent function, which can be viewed as a representation with perfect time resolution but no direct information about the signal’s frequency content. The other standard signal representation is a frequency spectrum, which is obtained by the Fourier transform of the signal and shows the magnitudes of the individual frequencies that make up the original signal but does not provide any information about the time instants when they occurred in the signal [1,2]. The Fourier transform definition states that the entire signal should be known up to infinity; furthermore, for their exhaustive representation, signals should be stationary, meaning that the frequency content should not change over time.

The definitions of the Fourier transform and its inverse are, respectively:

(1a) $$X\left(f\right)=F\left\{x\left(t\right)\right\}=\int_{-\infty }^{\infty }x\left(t\right)e^{-j2\pi ft}dt,$$

(1b) $$x\left(t\right)=F^{-1}\left\{X\left(f\right)\right\}=\int_{-\infty }^{\infty }X\left(f\right)e^{j2\pi ft}df,$$

where x(t) is the original time signal, X(f) is the Fourier transform of the original time-domain signal, *t* is the time variable, *f* is the frequency variable, and F and $F^{-1}$ are the Fourier transform and its inverse, respectively.

On the other hand, time–frequency (TF) analysis is an approach to signal analysis that is used to simultaneously track the signal’s time and frequency changes. The problem caused by finite signal duration and non-stationarity can be overcome by considering the original signal inside finite time windows, i.e., calculating the basic time–frequency representation known as the short-time Fourier transform (STFT). The time window acts as a filter that gives, as a result, the Fourier transform for the signal centered around the output time, which is the Fourier transform for that specific windowed data segment. The signal in each time window is assumed to be stationary and of known duration. Thus, the STFT may be defined as the Fourier transform of the original signal multiplied by a sliding window:

(2) $$F_{x}\left(t,f\right)=\int_{-\infty }^{\infty }x\left(\tau \right)h\left(\tau -t\right)e^{-j2\pi f\tau }d\tau ,$$

where $F_{x}\left(t,f\right)$ is the STFT and h(t) is the moving time window.

**Figure 1:**
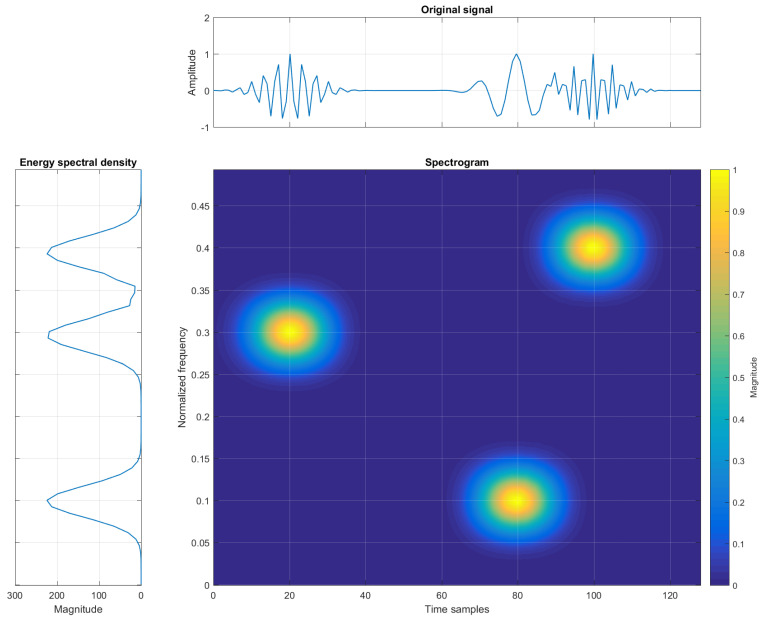
Example of three elementary Gaussian atoms in time, frequency, and TF domains (spectrogram).

Insight into both time and frequency domains is provided by TF signal analysis. The original signal, acquired in the time domain, is now represented in order to show time instants of frequency changes, allowing appropriate monitoring and tracking of non-stationary signals. An example of a signal in the time, frequency, and TF domains is shown in Figure 1.

There are several groups of time–frequency representations or distributions (TFRs/TFDs) to choose from, depending on the application. The three most important are the Cohen’s, affine, and reassigned classes. Cohen’s class is covariant to time and frequency shifts of the signal [3], while the affine class is covariant to time shifts or translations and the scaling or, rather, the dilatations of the signal, also called timescale representations or distributions. The most common example of the affine representation is the continuous wavelet transform, which linearly expands the signal to a set of analysis functions called wavelets that can be time-shifted or scaled appropriately to fit the original signal [4]. In some cases, a TFR can belong to both the Cohen’s and affine classes, depending on the properties it satisfies. Both contain bilinear or quadratic representations, and this non-linearity results in the presence of interference terms, also called cross-terms, in the TF plane [5]. These phenomena can complicate the interpretation of the representation by overlapping the signal components or producing negative values. Nevertheless, bilinear representations generally exhibit improved TF concentration and resolution, despite the interference terms being introduced [6,7].

#### 1.1.1. Analytic Form of a Signal

The first step in reducing the number of interferences in the TFR is using the analytic form of the signal. The analytic form gives a complex signal containing only the positive frequencies present in the original real-valued signal (the real part of the analytical signal corresponds to the original signal, while the imaginary part corresponds to the Hilbert transform of the original signal [8]). The spectrum of the analytic signal for f<0 is zero, while for f>0, the spectrum coincides with that of the real-valued signal [9]. By using an analytic function instead of a real-valued one, there is no loss of information caused by the removal of negative frequencies, since the Fourier transform satisfies Hermitian symmetry $X^{*}\left(f\right)=X\left(-f\right)$. Hence, most TFRs should be used with analytic forms of signals, constructed as in [10]:

(3a) $$x_{a}\left(t\right)=x\left(t\right)+jH\left\{x\left(t\right)\right\},$$

(3b) $$X_{a}\left(f\right)=2U\left(f\right)X\left(f\right)=X\left(f\right)+sgn\left(f\right)X\left(f\right),$$

where $x_{a}\left(t\right)$ is the analytic form of the signal, x(t) is the real-valued signal, H is the Hilbert transform, $X_{a}\left(f\right)$ is the spectrum of the analytic signal, X(f) is the original spectrum of the real-valued signal, and U(f) is the unit step function.

#### 1.1.2. Instantaneous Frequency

The purpose of the instantaneous frequency (IF) estimation is to capture and trace the frequency content resulting from non-stationarities of the real-valued signal or its analytic associate. It is defined as the derivative of the signal phase showing the time change in the dominant frequency [10,11,12]. Thus, for mono-component signals, the IF describes the behavior of the signal frequency over time [9,13].

For an analytic signal defined with time-varying amplitude a(t) and phase φ(t) defined as $x_{a}\left(t\right)=a\left(t\right)e^{j\varphi (t)}$, the IF can then be calculated as follows:

(4) $$f_{x}\left(t\right)=\frac{1}{2\pi }\frac{d\varphi (t)}{dt},$$

where φ(t) is the phase of the signal and $f_{x}\left(t\right)$ is the IF of the signal.

This IF estimation approach is not suitable for multi-component signals, especially when spectral components overlap in the TF plane.

#### 1.1.3. Group Delay

The group delay is dual to the IF and is defined as the derivative of the phase spectrum or frequency behavior as a function of time. It is also usually described as the time delay or average arrival time for a given frequency [9,13].

The Fourier transform for an analytic signal is defined with spectral amplitude B(f) and spectral phase ψ(f) as $X\left(f\right)=B\left(f\right)e^{j\psi (f)}$, and the group delay can be defined as:

(5) $$t_{x}\left(f\right)=-\frac{1}{2\pi }\frac{d\psi (f)}{df},$$

where ψ(f) is the spectral phase of the Fourier transform of the signal and $t_{x}\left(f\right)$ is the group delay (which is a function of frequency).

The IF and group delay can be approximated as inverse functions of each other for signals with large time-bandwidth products (T×B) [1,9].

This paper aims to provide comprehensive insight, including a comparison between TFRs from three classes: the Cohen’s, affine, and reassigned classes. We provide the theoretical basis behind the analyzed TFRs and showcase their behavior in noisy environments for different noise levels. We analyze TFRs’ performance for synthetic signals, both mono-component and multi-component, as well as on real-life examples. To the best of our knowledge, based on an extensive literature review, there exist no similar studies investigating the given TFRs from these three classes based on the accuracy in IF estimation, distribution concentration measured by entropy measures, and computational cost. The study provides support in selecting the appropriate TFR for a signal of interest being analyzed.

The rest of the paper is structured as follows. Section 2 provides insight into the theory behind the TFRs, explains the different types of TFRs, and lists some of the previous work in the field. Section 3 presents the application of TFRs to synthetic and real-life signals and compares TFR performance, providing details on the computational cost of the tested TFRs. Next, the obtained results are discussed and elaborated on in Section 4. Finally, the paper closes with concluding remarks in Section 5.

## 2. Affine TFRs with Respect to Cohen’s and Reassigned TFRs

The affine class is a group of bilinear representations that are covariant to time shifts and dilations of the signal [14] and it is the counterpart to Cohen’s class or the Weyl-Heisenberg group [14,15,16]. The time shift is the same covariance as in Cohen’s class, and the scale or dilation of the signal is defined as the reciprocal of the frequency [17,18]. Affine representations are suited for applications such as radar, sonar, self-similar signal analysis, multi-resolution signal analysis, and adaptive signal analysis [10,19,20]. Bilinearity results from the way the observed signal is compared to a delayed copy of itself by multiplication (autocorrelation) [9].

The general bilinear energy representation can be written as:

(6) $$\Omega_{x}\left(t,f\right)=\int_{-\infty }^{\infty }\int_{-\infty }^{\infty }K\left(t_{1},t_{2};t,f\right)x\left(t_{1}\right)x^{*}\left(t_{2}\right)dt_{1}dt_{2},$$

where $\Omega_{x}$ is the TFR, ν is the frequency shift, also known as Doppler, τ is the time delay or time shift, the product $x\left(t_{1}\right)x^{*}\left(t_{2}\right)$ is the useful part of the signal, and $K(t_{1},t_{2};t,f)$ is the arbitrarily parametrized 4-dimensional kernel characterizing the TFR properties. The kernel $K(t_{1},t_{2};t,f)$ can also assume a different form, where $t_{1}$ and $t_{2}$ are replaced by $f_{1}$ and $f_{2}$, respectively (the different form is obtained by applying a double Fourier transform to the original kernel).

According to the energy conservation constraint, the parametrization kernel *K* must satisfy the following condition:

(7) $$\int_{-\infty }^{\infty }\int_{-\infty }^{\infty }K\left(t_{1},t_{2};t,f\right)dtdf=\delta \left(t_{1}-t_{2}\right),$$

where δ is the Dirac function.

The general affine representation form, which can be adapted to several representations by changing the index *k*, is defined as follows:

(8a) $$\Omega_{x}^{\left(k\right)}\left(t,f\right)=f\int_{-\infty }^{\infty }\mu_{k}\left(u\right)X\left(\lambda_{k}\left(u\right)f\right)X^{*}\left(\lambda_{k}\left(-u\right)f\right)e^{j2\pi ft\zeta_{k}\left(u\right)}du,$$

(8b) $$\lambda_{k}\left(u\right)={\left(k\frac{e^{-u}-1}{e^{-ku}-1}\right)}^{\frac{1}{k-1}},k\neq 0,1,$$

(8c) $$\lambda_{k=0}\left(u\right)=\frac{u}{1-e^{-u}},\lambda_{k=1}\left(u\right)=exp\left(1+\frac{ue^{-u}}{e^{-u}-1}\right),$$

(8d) $$\zeta_{k}\left(u\right)=\lambda_{k}\left(u\right)-\lambda_{k}\left(-u\right),$$

where $\mu_{k}\left(u\right)$ is a continuous positive weighting function, the choice of which affects the representation properties, and *u* is the dual variable of the product t×f.

Changing the value of the index *k* in $\lambda_{k}\left(u\right)$ and $\zeta_{k}\left(u\right)$, we obtain various representations [19,20], including the following:

- k=2—Affine Wigner representation (extended covariance along straight line paths);
- k=1/2—D-Flandrin representation (extended covariance along square-root-hyperbolic paths);
- k=0—Bertrand representation (extended covariance along hyperbolic paths);
- k=−1—Unterberger representation;
- k=±5—Approximate affine Wigner representations (unsmoothed);
- k=±∞—Margenau–Hill representation.

Choosing different $\mu_{k}\left(u\right)$ functions for one specific value of *k* makes it possible to obtain different variations of the selected distribution, one of the examples being the active or passive Unterberger representation.

### 2.1. Kernels

Kernels result from arbitrary parametrizations that characterize the properties of the representation. By restricting the structure of the parametrized kernel and specifying certain analytic properties that the representations must satisfy, subclasses can be identified [17].

Kernels can be defined as:

(9a) $$\phi_{t-f}\left(t,f\right)=\delta \left(t\right)\delta \left(f-f_{0}\right),$$

(9b) $$\phi_{d-D}\left(\tau ,\nu \right)=\int_{-\infty }^{\infty }\phi_{t-d}\left(t,\tau \right)e^{-j2\pi \nu t}dt=\int_{-\infty }^{\infty }\int_{-\infty }^{\infty }\phi_{t-f}\left(t,f\right)e^{j2\pi (f\tau -\nu t)}dfdt,$$

(9c) $$\phi_{f-D}\left(f,\nu \right)=\int_{-\infty }^{\infty }\phi_{t-f}\left(t,f\right)e^{-j2\pi \nu t}dt=\int_{-\infty }^{\infty }\int_{-\infty }^{\infty }\phi_{t-d}\left(t,\tau \right)e^{-j2\pi (t\nu +\tau f)}dtd\tau ,$$

(9d) $$\phi_{t-f}\left(t,f\right)=\int_{-\infty }^{\infty }\phi_{t-d}\left(t,\tau \right)e^{-j2\pi f\tau }d\tau =\int_{-\infty }^{\infty }\phi_{f-D}\left(f,\nu \right)e^{j2\pi \nu t}d\nu ,$$

where $\phi_{t-d}\left(t,\tau \right)$ is the time–delay kernel, $\phi_{d-D}\left(\tau ,\nu \right)$ is the delay–Doppler kernel, $\phi_{f-D}\left(f,\nu \right)$ is the frequency–Doppler kernel, $\phi_{t-f}\left(t,f\right)$ is the time–frequency kernel, δ is the Dirac delta function, and $f_{0}$ is the arbitrary non-zero frequency (usually set to 1 Hz).

The different kernels, representing the various domains, and the Fourier transform between them can be represented using a commutative diagram (10) that explains their mutual relationships [9,21,22]:
(10)
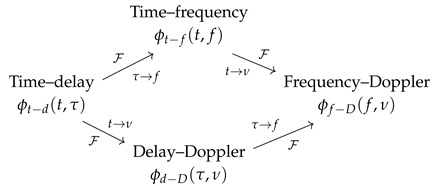


The time–delay kernel is mainly used to compute the representation from the time-domain signal, while the delay–Doppler kernel allows a simpler analysis of the characteristics of the representation. The time–frequency kernel can be interpreted as a two-dimensional low-pass filter that smooths the signal in both the time and frequency domains [8,23].

Depending on the number of variables, the kernel can be one-dimensional, two-dimensional, or higher-dimensional. The kernel can be independent on a specific variable. Separable kernels consist of the product of two windowing functions, each one for its respective domain. These windowing functions can be set independently of each other, offering the possibility of independent resolution tuning for each domain [9,10,21,24].

An example of a separable kernel consisting of two windowing functions may be defined as follows:

(11) $$\phi \left(\nu ,\tau \right)=G_{1}\left(\nu \right)g_{2}\left(\tau \right).$$

This form of the kernel can be transferred to the time–frequency domain by applying the inverse and forward Fourier transforms to the two windows, respectively $\Phi \left(t,f\right)=F^{-1}\left\{G_{1}\left(\nu \right)\right\}F\left\{g_{2}\left(\tau \right)\right\}=g_{1}\left(t\right)G_{2}\left(f\right)$.

The separable kernel ensures that the 2D convolution is replaced by two 1D convolutions, and so the representation becomes:

(12) $$\Omega_{x}^{\Phi }\left(t,f\right)=g_{1}\left(t\right)*\Omega_{x}\left(t,f\right)*G_{2}\left(f\right).$$

If $G_{1}\left(\nu \right)=1$, then $\phi \left(\nu ,\tau \right)=g_{2}\left(\tau \right)$, and the kernel becomes Doppler independent with smoothing only in the frequency direction. In that case, the resulting representation is:

(13) $$\Omega_{x}^{\Phi }\left(t,f\right)=\Omega_{x}\left(t,f\right)*G_{2}\left(f\right).$$

If $g_{2}\left(\tau \right)=1$, then $\phi \left(\nu ,\tau \right)=G_{1}\left(\nu \right)$, and the kernel becomes delay independent with smoothing only in the time direction, and the representation becomes:

(14) $$\Omega_{x}^{\Phi }\left(t,f\right)=g_{1}\left(t\right)*\Omega_{x}\left(t,f\right).$$

### 2.2. Energy

The signal energy is defined as the squared modulus of the signal in the time or frequency domain, according to Parseval’s theorem [8,25]:

(15) $$E_{x}=\int_{-\infty }^{\infty }{\left|x\left(t\right)\right|}^{2}dt=\int_{-\infty }^{\infty }{\left|X\left(f\right)\right|}^{2}df,$$

where the energy representations are the instantaneous power ${\left|x\left(t\right)\right|}^{2}$ for the time domain and the spectral energy density ${\left|X\left(f\right)\right|}^{2}$ for the frequency domain.

The bilinear TFRs are signal energy distributions defined as the combination of the instantaneous power and the spectral energy density [17]:

(16) $$E_{x}=\int_{-\infty }^{\infty }\int_{-\infty }^{\infty }\Omega_{x}\left(t,f\right)dtdf.$$

The representation $\Omega_{x}$ satisfies the energy conservation property for the affine class only if the kernel satisfies the following condition:

(17) $$\int_{-\infty }^{\infty }\frac{\phi_{f-D}\left(0,\nu \right)}{\left|\nu \right|}d\nu =1.$$

By integrating the representation over the timescale plane, one obtains the signal energy [17].

### 2.3. Covariance

The covariance of the affine class refers to time shifts and scale changes in the signal. This property is based on the parameters of the affine group denoted by A(α,τ), where α stands for the analyzing scale parameter and τ for the time shift. For the one-dimensional affine group, the group operation is (A,B)(α,τ)=(Aα,B+Aτ), corresponding to a time axis clock change with t→αt+τ, where the scale parameter α is defined as $\alpha =\frac{f_{0}}{f}$, and the arbitrary non-zero frequency is $f_{0}=1\;\mathrm{Hz}$ [17].

Thus, the affine group of transforms for a time signal and its frequency counterpart are:

(18a) $$x\left(t\right)\to x_{\alpha ,\tau }\left(t\right)=\frac{1}{\sqrt{\alpha }}x\left(\frac{t-\tau }{\alpha }\right),$$

(18b) $$X\left(f\right)\to X_{\alpha ,\tau }\left(f\right)=\sqrt{\alpha }e^{-j2\pi f\tau }X\left(\alpha f\right).$$

The covariance requirement for the representation can then be expressed as:

(19) $$\Omega_{x_{\alpha ,\tau }}\left(t,f\right)=\Omega_{x}\left(\frac{t-\tau }{\alpha },\alpha f\right).$$

An example of an affine covariant representation is the convolution of the Wigner representation, which is a member of the Cohen class, with a signal-independent kernel as:

(20) $$\Omega_{x}\left(t,f\right)=\int_{-\infty }^{\infty }\int_{-\infty }^{\infty }\Pi \left(\frac{t-\tau }{\alpha },\alpha f\right)W_{x}\left(t,f\right)dtdf,$$

where $W_{x}\left(t,f\right)$ is the Wigner representation and Π(t,f) is the signal-independent kernel, which can also be denoted as the time–frequency kernel $\phi_{t-f}\left(t,f\right)$. The representation is called the pseudo-affine Wigner representation.

### 2.4. Marginals

The marginal constraint states that the integral of the representation over one variable gives the energy corresponding to the other variable [25,26].

The marginal properties are defined for both the time and frequency domains as follows:

(21a) $$\int_{-\infty }^{\infty }\Omega_{x}\left(t,f\right)dt={\left|X\left(f\right)\right|}^{2},$$

(21b) $$\int_{-\infty }^{\infty }\Omega_{x}\left(t,f\right)df={\left|x\left(t\right)\right|}^{2}.$$

Satisfying the marginal conditions implies the conservation of the representation energy [27,28]. Structural constraints on the kernel assure that the marginals are satisfied [17]. These constraints refer to the marginal distribution in frequency (energy spectrum density) and to the marginal distribution in time (instantaneous power), as follows:

(22a) $$\phi_{f-D}\left(0,\nu \right)=\delta \left(\nu -1\right),$$

(22b) $$\int_{-\infty }^{\infty }\phi_{d-D}\left(f\tau ,\frac{\nu }{f}\right)df=\delta \left(\tau \right).$$

### 2.5. Interference Terms

Due to the bilinear nature of the affine class, the interference terms (also known as cross-terms) are generated at midway points between the original components (also called auto-terms) or between other interference terms [29]. Thus, due to the bilinearity, the sum of the representations of two signals is not equal to the representation of their sum [2,9,30].

Interference terms oscillate with a frequency that is proportional to the distance of the auto-terms [13,21]. The direction of the oscillations is orthogonal to the line connecting the auto-terms; the number of interference terms is n(n−1)/2, where *n* is the number of signal components.

For a two component signal, $x\left(t\right)=x_{1}\left(t\right)+x_{2}\left(t\right)$, the bilinear representation is:

(23a) $$\Omega_{x}\left(t,f\right)=\Omega_{x1}\left(t,f\right)+\Omega_{x2}\left(t,f\right)+2ℜ\left[\Omega_{x1,2}\left(t,f\right)\right],$$

(23b) $$2ℜ\left[\Omega_{x1,2}\left(t,f\right)\right]=2ℜ\left[\Omega_{x1,x2}\left(t,f\right)+\Omega_{x2,x1}\left(t,f\right)\right]=2ℜ\left[F\left(x_{1}\left(t\right)x_{2}^{*}\left(t\right)\right)\right],$$

where $\Omega_{x1}\left(t,f\right)$ and $\Omega_{x2}\left(t,f\right)$ are the auto-terms, and $2ℜ\left[\Omega_{x1,2}\left(t,f\right)\right]$ is the cross-term.

**Figure 2:**
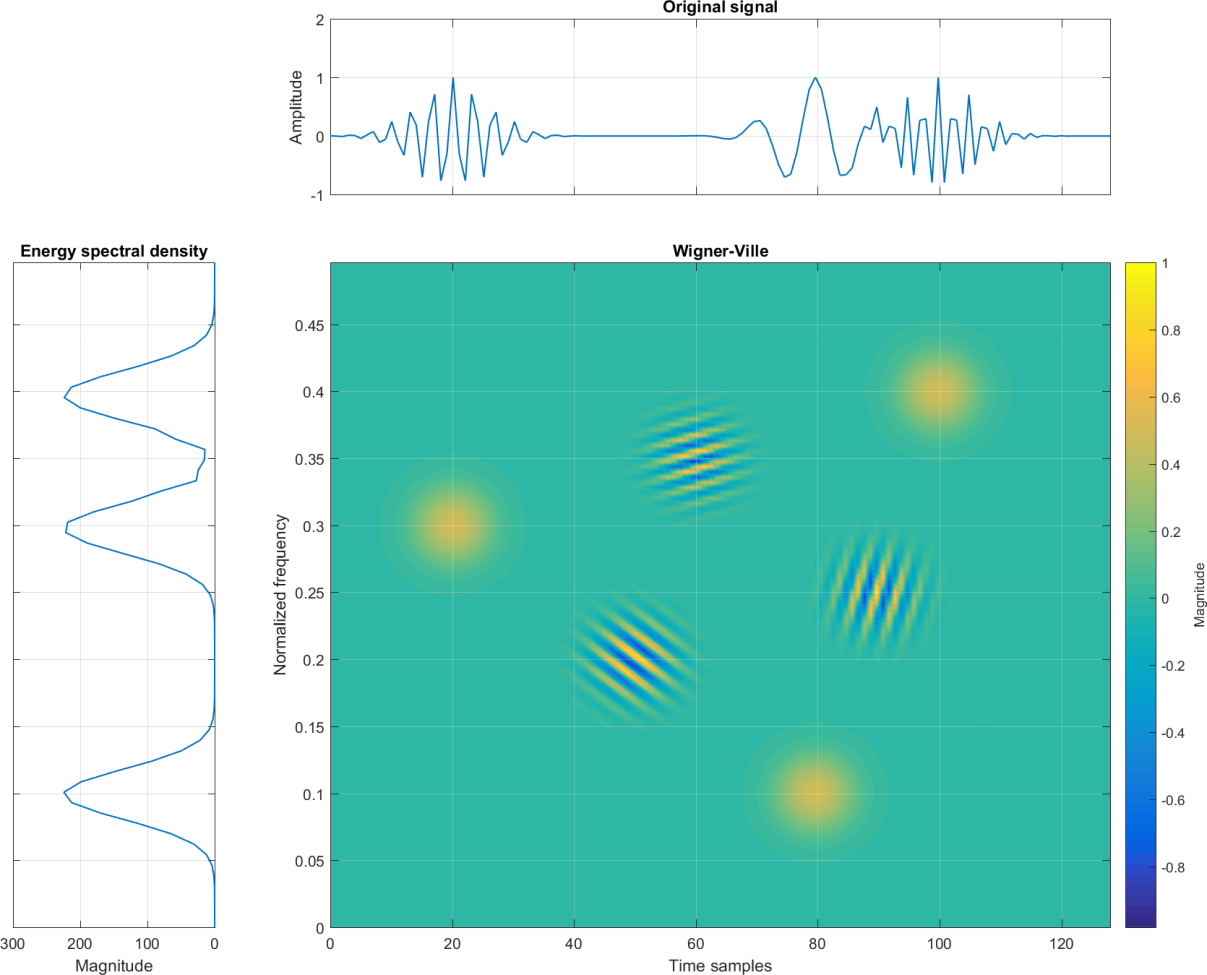
Example of three elementary Gaussian atoms in the time, frequency, and TF domains (Wigner-Ville) with interferences.

Figure 2 reports a TFR with cross-terms being generated between the three auto-terms. The location of the cross-term for a two-component signal is defined at the midpoint between the auto-terms, or at the midpoints of the two coordinates defined as the mean values $t_{i}=\frac{t_{1}+t_{2}}{2}$ and $f_{i}=\frac{f_{1}+f_{2}}{2}$, for time and frequency coordinates, respectively [31].

### 2.6. Reassignment Method

This approach was first defined by Kodera et al. [32,33] under the name “Modified Moving Window Method”. Although the method was defined only for spectrograms and could not be applied to discrete signals because of the partial derivatives used in the method’s definition, it was later extended by Auger and Flandrin [34,35], generalized for any bilinear time–frequency or timescale representation, and called the “reassignment method”.

The reassignment method is a local post-processing method for signal representations, with the goal of improving the signal sharpness or concentration, and it refocuses the energy distribution for better readability in the TF plane. It is applicable to the Cohen’s and affine classes as well as any bilinear representation, improving the TF localization of the signal and suppressing the interference of the cross-terms. This method is the second of a two-step procedure in which a 2D low-pass filter smoothing kernel is first applied to reduce the interfering components, which also leads to the smearing of the signal in the *t*–*f* plane. The second step is squeezing, which refocuses the signal terms after the smoothing procedure [36].

Different researchers introduced this method under different names, such as the method of reassignment, remapping, *t*–*f* reassignment, and the modified moving window method. Unlike other representations, this method requires the signal phase and not just the magnitude. The phase is needed because the components that represent the maximum contribution to the signal, also known as the center of gravity, have a slow phase variation through time and can thus be detected by observing the rate of variation of the phase [37].

The reassignment is performed by mapping the computed data points from their initial coordinates (t,f) closer to the true region of the signal support [14,38], also known as the centroid or center of gravity $\left(\hat{t}\left(t,f\right),\hat{f}\left(t,f\right)\right)$. The center of gravity is calculated for each windowed region of the *t*–*f* plane, and then the calculated energy values for that region are placed at that point, rather than at the geometric center [13,39]. By averaging the energy given by the windowed region and then assigning the value to the center of gravity for that region, the energy is focused where it actually occurred [33].

The definitions of the center of gravity coordinates for time and frequency, respectively, are:

(24a) $$\hat{t}\left(t,f\right)=t-\frac{1}{2\pi }\frac{\partial \varphi (t,f)}{\partial f}=-\frac{1}{2\pi }\frac{\partial \varphi (t,f)}{\partial f},$$

(24b) $$\hat{f}\left(t,f\right)=\frac{1}{2\pi }\frac{\partial \varphi (t,f)}{\partial t}=f+\frac{1}{2\pi }\frac{\partial \varphi (t,f)}{\partial t},$$

where $\hat{t}\left(t,f\right)$ and $\hat{f}\left(t,f\right)$ are the coordinates of the center of gravity and can also be referred to as local group delay and local IF, respectively, while φ(t,f) is the phase of the signal. The generalized form for Cohen’s class is then defined as:

(25a) $$\hat{t}\left(t,f\right)=t-\frac{\int_{-\infty }^{\infty }\int_{-\infty }^{\infty }\tau \phi \left(\tau ,\nu \right)W_{x}\left(t-\tau ,f-\nu \right)d\tau d\nu }{\int_{-\infty }^{\infty }\int_{-\infty }^{\infty }\phi \left(\tau ,\nu \right)W_{x}\left(t-\tau ,f-\nu \right)d\tau d\nu },$$

(25b) $$\hat{f}\left(t,f\right)=f-\frac{\int_{-\infty }^{\infty }\int_{-\infty }^{\infty }\nu \phi \left(\tau ,\nu \right)W_{x}\left(t-\tau ,f-\nu \right)d\tau d\nu }{\int_{-\infty }^{\infty }\int_{-\infty }^{\infty }\phi \left(\tau ,\nu \right)W_{x}\left(t-\tau ,f-\nu \right)d\tau d\nu },$$

(25c) $$R_{C}\left(t^{\prime },f^{\prime }\right)=\int_{-\infty }^{\infty }\int_{-\infty }^{\infty }TFR\left(t,f\right)\delta \left[t^{\prime }-\hat{t}\left(t,f\right)\right]\delta \left[f^{\prime }-\hat{f}\left(t,f\right)\right]dtdf,$$

where ϕ(τ,ν) is the kernel, $W_{x}$ is the Wigner–Ville representation, TFR(t,f) is the representation being reassigned, δ is the Dirac delta function, $R_{C}\left(t^{\prime },f^{\prime }\right)$ is the reassigned representation, and $t^{\prime }$ and $f^{\prime }$ are the coordinates of any point.

The generalized form for the affine class is then defined as:

(26a) $$\hat{t}\left(t,\alpha \right)=t-\frac{\int_{-\infty }^{\infty }\int_{-\infty }^{\infty }\tau \phi \left(\frac{\tau }{\alpha },f_{0}-\alpha \nu \right)W_{x}\left(t-\tau ,\nu \right)d\tau d\nu }{\int_{-\infty }^{\infty }\int_{-\infty }^{\infty }\phi \left(\frac{\tau }{\alpha },f_{0}-\alpha \nu \right)W_{x}\left(t-\tau ,\nu \right)d\tau d\nu },$$

(26b) $$\hat{f}\left(t,\alpha \right)=\frac{f_{0}}{\hat{\alpha }\left(t,\alpha \right)}=\frac{\int_{-\infty }^{\infty }\int_{-\infty }^{\infty }\nu \phi \left(\frac{\tau }{\alpha },f_{0}-\alpha \nu \right)W_{x}\left(t-\tau ,\nu \right)d\tau d\nu }{\int_{-\infty }^{\infty }\int_{-\infty }^{\infty }\phi \left(\frac{\tau }{\alpha },f_{0}-\alpha \nu \right)W_{x}\left(t-\tau ,\nu \right)d\tau d\nu },$$

(26c) $$R_{A}\left(t^{\prime },\alpha^{\prime }\right)=\int_{-\infty }^{\infty }\int_{-\infty }^{\infty }{\left(\alpha^{{}^{\prime }}\right)}^{2}TSR\left(t,\alpha \right)\delta \left[t^{\prime }-\hat{t}\left(t,\alpha \right)\right]\delta \left[\alpha^{\prime }-\hat{\alpha }\left(t,\alpha \right)\right]dt\frac{d\alpha }{\alpha^{2}},$$

where $\alpha =\frac{f_{0}}{f}$, $f_{0}=1\;\mathrm{Hz}$, TSR(t,α) is any timescale representation, $R_{A}\left(t^{\prime },\alpha^{\prime }\right)$ is the reassigned representation, and $\alpha^{\prime }$ is the scale coordinate for any point.

However, the reassignment method encounters performance difficulties for noisy signals with a low signal-to-noise ratio (SNR), where the computed centers of gravity might not actually be part of the signal due to the noise; they could instead be assigned to random noise pattern locations [40]. In addition, representations resulting from the application of the reassignment method may not satisfy some properties that the original representations satisfied, e.g., energy preservation. The reassigned distribution is time and frequency shift covariant, although it is no longer a bilinear representation [34,41].

### 2.7. Affine Class Distributions

Several TFR methods can be assigned to the affine class. In the following subsections, we list some of them with their definitions and main characteristics.

#### 2.7.1. Scalogram

The scalogram is obtained by squaring the continuous wavelet transform magnitudes or, rather, by frequency-dependent affine smoothing of the Wigner representation with the analyzing wavelet. It provides the energy distribution of the signal in the TF plane and represents the simplest affine TFR. Its main drawback is poor resolution due to the proportional-bandwidth time–frequency trade-off that is controlled by the analyzing wavelet. Thus, it is impossible to control the time and frequency resolutions independently. However, it has fewer of the interference terms that disturb other affine class TFRs because they are restricted only to those regions where the signal terms overlap [13,20].

The scalogram can be defined as a 2D kernel windowing of the Wigner representation [16] or as the absolute square of the continuous wavelet transform:

(27) $$\begin{array}{cc} S_{x}\left(t,f\right) & =\int_{-\infty }^{\infty }\int_{-\infty }^{\infty }W_{x}\left(\tau ,\nu \right)\phi_{t-f}\left(\frac{\tau -t}{\alpha },\alpha \nu \right)d\tau d\nu \end{array}$$

(28) $$\begin{array}{cc} \; & \;\;={\left|CWT_{x}\left(t,f\right)\right|}^{2}=\frac{1}{\left|\alpha \right|}{\left|\int_{-\infty }^{\infty }x\left(t\right)h\left(\frac{t-\tau }{\alpha }\right)dt\right|}^{2}, \end{array}$$

where $S_{x}\left(t,f\right)$ is the scalogram, $W_{x}\left(t,f\right)$ is the Wigner representation, $\phi_{t-f}\left(t,f\right)$ is the time–frequency kernel, $CWT_{x}\left(t,f\right)$ is the continuous wavelet transform, $\alpha =\frac{f_{0}}{f}$, and h(t) is the mother wavelet.

#### 2.7.2. Smoothed Pseudo-Affine Wigner Distribution

The smoothed pseudo-affine Wigner distribution (SPAWD) shows similar properties to the wavelet transform; however, it exhibits a higher time–frequency resolution. Its two main drawbacks are the interference terms generated due to bilinearity and the difficult application to long-duration time signals. The method uses a short-time window that controls the trade-off between interference attenuation and resolution, making its computation more efficient. A transition from the high-resolution affine Wigner representation with interferences to the interference-free scalogram is possible. The SPAWD is defined as a self-correlation of the wavelet transform across frequency; the time windowing suppresses the interference components in the frequency direction, while the time direction smoothing is implemented by convolving the time windowing with a low-pass function [42]. Windowing must be frequency-dependent so that the resulting TFD remains affine covariant; thus, the smoothing in the frequency direction exhibits proportional bandwidth rather than constant bandwidth [13].

The enhancement in resolution is the result of the self-correlation that is applied to the signal, behaving like match-filtering, rather than just squaring the signal as for the wavelet transform [13,20]. The algorithm for the SPAWD is defined by computing the wavelet transform of the signal and by performing the generalized frequency correlation for each time point, which is efficiently implemented by the Mellin transform.

The general representation and the representation parameters (that for k=2 result in the affine smoothed pseudo-Wigner–Ville representation) are defined as follows:

(29a) $$\begin{array}{cc} {\tilde{P}}_{x}^{k}\left(t,f\right) & =f\int_{-\infty }^{\infty }\mu_{k}\left(u\right)X\left(f\lambda_{k}\left(u\right)\right)X^{*}\left(f\lambda_{k}\left(-u\right)\right)e^{j2\pi ft\zeta_{k}\left(u\right)}du \end{array}$$

(29b) $$\begin{array}{cc} \; & \;=f\int_{-\infty }^{\infty }G\left(u\right)\frac{\mu_{k}\left(u\right)}{\sqrt{\lambda_{k}\left(u\right)\lambda_{k}\left(-u\right)}}\tilde{CWT_{x}^{\psi }}\left(t,\lambda_{k}\left(u\right)f\right){\left[\tilde{CWT_{x}^{\psi }}\left(t,\lambda_{k}\left(-u\right)f\right)\right]}^{*}du, \end{array}$$

(29c) $$\lambda_{k=2}\left(u\right)=1+\tanh\left(\frac{u}{2}\right),$$

(29d) $$\mu_{k=2}\left(u\right)=1-\tanh^{2}\left(\frac{u}{2}\right),$$

(29e) $$\zeta_{k=2}\left(u\right)=2\tanh\left(\frac{u}{2}\right),$$

where ${\tilde{P}}_{x}^{k}\left(t,f\right)$ is the smoothed pseudo-affine Wigner representation, $\tilde{CWT_{x}^{\psi }}\left(t,f\right)$ is the time–frequency version of the continuous wavelet transform with a band pass wavelet function $\psi \left(\tau \right)=h\left(\tau \right)e^{j2\pi \tau }$, $\lambda_{k}\left(u\right)$ as per Equation (8b), and G(u) is the dual variable window or low-pass function that smooths the TFR by proportional-bandwidth time smoothing.

#### 2.7.3. Unitary Bertrand Distribution

Also known as the $P_{0}$ representation, obtained for k=0, its role in the affine class is comparable to the Wigner representation for the Cohen class. The unitary Bertrand representation localizes hyperbolas on the time–frequency plane, making it a hyperbolic representation [17,22,31].

The Bertrand representation $B_{x}\left(t,f\right)$ and its parameters for k=0 are defined as follows:

(30a) $$B_{x}\left(t,f\right)=f\int_{-\infty }^{\infty }\mu_{k}\left(u\right)X\left(f\lambda_{k}\left(u\right)\right)X^{*}\left(f\lambda_{k}\left(-u\right)\right)e^{j2\pi ftu}du,$$

(30b) $$\lambda_{k=0}\left(u\right)=\frac{e^{\frac{u}{2}}\frac{u}{2}}{\sinh(\frac{u}{2})},$$

(30c) $$\mu_{k=0}\left(u\right)=\sqrt{\lambda_{k=0}\left(u\right)\lambda_{k=0}\left(-u\right)}=\frac{\frac{u}{2}}{\sinh(\frac{u}{2})}.$$

By using $\mu_{k=0}\left(u\right)=1$, we obtain the nonunitary Bertrand representation instead.

#### 2.7.4. Unterberger Distribution

The Unterberger representation, preserving the scaling properties across frequency while smoothing the interferences in the frequency direction, is defined for the value k=−1 in Equation (8). There are two forms of the representation: the active form that exhibits the localization property and the passive form that does not [20]. The active Unterberger representation localizes on squared hyperbolas.

The representation and its parameters are defined as follows:

(31a) $$\begin{array}{cc} U_{x}^{a}\left(t,f\right) & =f\int_{-\infty }^{\infty }\mu_{k}\left(u\right)X\left(f\lambda_{k}\left(u\right)\right)X^{*}\left(f\lambda_{k}\left(-u\right)\right)e^{j2\pi ft\zeta_{k}\left(u\right)}du \end{array}$$

(31b) $$\begin{array}{cc} \; & \;=f\int_{-\infty }^{\infty }\left(1+\frac{1}{\gamma^{2}}\right)X\left(f\gamma \right)X^{*}\left(\frac{f}{\gamma }\right)e^{j2\pi ft\left(\gamma -\frac{1}{\gamma }\right)}d\gamma , \end{array}$$

(31c) $$\mu_{k=-1}\left(u\right)=\cosh\left(\frac{u}{2}\right),$$

(32a) $$\begin{array}{cc} U_{x}^{p}\left(t,f\right) & =f\int_{-\infty }^{\infty }\mu_{k}\left(u\right)X\left(f\lambda_{k}\left(u\right)\right)X^{*}\left(f\lambda_{k}\left(-u\right)\right)e^{j2\pi ft\zeta_{k}\left(u\right)}du \end{array}$$

(32b) $$\begin{array}{cc} \; & =\int_{-\infty }^{\infty }\frac{2}{\gamma }X\left(f\gamma \right)X^{*}\left(\frac{f}{\gamma }\right)e^{j2\pi ft\left(\gamma -\frac{1}{\gamma }\right)}d\gamma , \end{array}$$

(32c) $$\lambda_{k=-1}\left(u\right)=\gamma \left(u\right)=e^{\frac{u}{2}},$$

(32d) $$\zeta_{k=-1}\left(u\right)=2\sinh\left(\frac{u}{2}\right),$$

where $U_{x}^{a}\left(t,f\right)$ is the active Unterberger representation and $U_{x}^{p}\left(t,f\right)$ is the passive Unterberger representation, with their respective parameters. The active Unterberger representation is achieved by using a specific $\mu_{k=-1}\left(u\right)$ function, as defined in Equation (31c), opposed to the passive Unterberger representation.

#### 2.7.5. D-Flandrin Distribution

The D-Flandrin representation perfectly localizes signals on square root hyperbolas [31], and it is obtained for the value $k=\frac{1}{2}$.

The D-Flandrin representation $D_{x}\left(t,f\right)$ and its parameters are defined as follows:

(33a) $$\begin{array}{cc} D_{x}\left(t,f\right) & =f\int_{-\infty }^{\infty }\mu_{k}\left(u\right)X\left(f\lambda_{k}\left(u\right)\right)X^{*}\left(f\lambda_{k}\left(-u\right)\right)e^{j2\pi ft\zeta_{k}\left(u\right)}du \end{array}$$

(33b) $$\begin{array}{cc} \; & \;=f\int_{-\infty }^{\infty }\left(1-{\left(\frac{\gamma }{4}\right)}^{2}\right)X\left(f{\left[1+\frac{\gamma }{4}\right]}^{2}\right)X^{*}\left(f{\left[1-\frac{\gamma }{4}\right]}^{2}\right)e^{j2\pi ft\gamma }d\gamma , \end{array}$$

(33c) $$\lambda_{k=\frac{1}{2}}\left(u\right)={\left[1+\tanh\left(\frac{u}{4}\right)\right]}^{2},$$

(33d) $$\zeta_{k=\frac{1}{2}}\left(u\right)=\gamma \left(u\right)=4\tanh\left(\frac{u}{4}\right),$$

(33e) $$\mu_{k=\frac{1}{2}}\left(u\right)=1-\tanh^{2}\left(\frac{u}{4}\right).$$

By using a function different from $\mu_{k=\frac{1}{2}}\left(u\right)$ as defined in Equation (33e), the D-Distribution representation is obtained instead [20,31].

#### 2.7.6. Other Affine Class Distributions

Affine time–frequency representations were first introduced in 1985 by Pierre Bertrand and Jaqueline Bertrand [43,44] soon after the wavelet theory was developed by Grossmann and Morlet [17]. The first affine representation was later referred to as the unitary affine Bertrand representation. In 1990, Flandrin and Rioul applied affine smoothing on some representations from Cohen’s class [45].

Flandrin and Rioul [45] provided a description of the affine smoothing of the Wigner–Ville distribution that resulted in a new class of representations with scale-dependent smoothing. The choices for the smoothing function and the properties were discussed. In addition, smoothing using separable Gaussian kernels was discussed, with the possibility of obtaining a continuous transition from spectrograms to scalograms, with the Wigner–Ville distribution between them. It was shown that it was possible to set up a specific requirement for a given application and then build a subset of timescale energy representations that meet those requirements by setting the values of selected parameters.

In [46], Ovarlez et al. provided more efficient algorithms based on the fast discrete Mellin transform for easier computation of the affine-group-affiliated TF distributions. Since the affine distributions use stretched forms of signals, they are normally more challenging to compute compared to standard techniques. Using only fast Fourier transform (FFT) routines made the algorithm very fast, allowing it to be employed as a practical tool for broad-band signal study.

Shenoy and Parks [47] proposed a symmetrized version of the wideband ambiguity function; by taking its 2D Fourier transform, the resulting function had properties similar to the Wigner distribution and was called the affine Wigner distribution. The introduced affine Wigner distribution was compared to the Wigner and the Q-distributions, and its properties were described. The method was based on group theory, and the results were compared to those presented in previous research by Altes, Rioul, and Flandrin.

Flandrin and Gonçalvès considered the geometry of bilinear affine distributions in the time–frequency plane [48]. The localization properties and generalized means of interference terms, as well as the generalized construction means, were established. It was stated that for frequency modulated (FM) signals, the defined general construction rules could be refined using the study of a critical manifold and stationary phase-type approximation in the case of point-wise application of those rules.

Flandrin and Gonçalvès [31] also considered the geometry of Bertrand’s bilinear affine distributions in the time–frequency plane. The localization properties, symmetry, and generalized means of interference terms, as well as the generalized construction means, were established. The reported theoretical results were corroborated by analytical and numerical examples. The affine distributions were shown to obey most of the same construction rules that the Wigner–Ville distribution obeys. A way of predicting interference diagrams was given for the affine class distributions and compared to the real interference patterns, showing good agreement between numerical computation and the real distribution generated for the signal.

Gonçalvès and Baraniuk suggested a set of pseudo- and smoothed pseudo-affine Wigner distributions [19]. When a short time window that controls the trade-off between localization and interference attenuation was applied to a pseudo-Wigner TFD, the pseudo-affine Wigner distribution was obtained. The applied windowing was frequency-dependent, so that the TFD remained affine, and as a result, the bandwidth was proportional rather than constant as in the pseudo-Wigner distribution. The pseudo-affine Wigner distribution was defined as the convolution of the wavelet transform with itself. The pseudo-affine version of the Wigner distribution can be interpreted as the sliding version of the affine Wigner distribution, thus making it suitable for long-duration signals and online real-time operation with the same computation cost as for the continuous wavelet transform. The introduced time windowing acted like a proportional bandwidth filter for the interference components oscillating in the frequency direction. A second window was utilized for smoothing in the time direction and for the suppression of interference components. This method provided continuous transition ability in smoothing from the affine Wigner distribution to the scalogram.

In [49], Murray et al. proposed a new higher-order Bertram distribution ($HO-P_{0}D$) as an extension of the Bertram distribution ($P_{0}$); it preserved scale changes as well as constant and hyperbolic time shifts of the signal. A class of smoothed higher-order Bertram distributions was also derived; a formulation was proposed for the higher-order extension of the quadratic class that preserved scale changes and constant time shifts. A novel higher-order distribution was proposed as an extension of the second-order Bertrand distribution, and a high-order affine class for multidimensional smoothing of a higher-order Wigner distribution was introduced.

In [20], Gonçalvès and Baraniuk introduced pseudo-affine Wigner distributions as tools for time-varying spectral analysis. These new timescale distributions demanded reduced computational power, allowing online operation with resource costs similar to the continuous wavelet transform. The distributions offered the suppression of interference terms because of proportional bandwidth smoothing due to the short-time window that controls localization. The wavelet-based structure for these distributions allowed continuous transition in smoothing from the scalogram to the affine Wigner distribution. An alternative set of generators was introduced for this class that simplified the kernel formulation and helped design new distributions for specific signal classes.

Iribarren et al. dealt in [50] with monitoring conditions and the prediction of faults on rotating machines by implementing the processing of non-stationary vibrations using the affine Wigner distribution. The synthesis of the technique was briefly explained. Tests were performed on synthetic and real-life signals and showed promising results in detecting non-stationary events. The diagnosed faults were characterized by complex spectrum changes, and weak vibration non-stationarities were used for the condition monitoring of rotating machines. The tested distributions were the STFT, the smoothed pseudo-Wigner–Ville distribution, and the SPAWD. It was concluded that the affine distribution presented higher resolution but often exhibited undesired interference. Nevertheless, the SPAWD presented fewer interference cross-terms than the SPWVD.

Murray et al. proposed in [18] a new higher-order affine TF representation called HO-TFR. The new class was of a higher order than quadratic TFRs (N>2). Five alternative formulations were provided that defined multidimensional smoothing kernels. The new higher class preserved the time shift, frequency shift, and signal scale. Another subclass was defined that crossed with Cohen’s class and satisfied three covariances with a higher-order affine–Cohen intersection. Simplified formulations for each member of the new higher-order affine–Cohen subclass were provided as one-dimensional functions.

Next, in [51], Gosme et al. proposed a method for adaptive and iterative smoothing of bilinear affine representations while preserving the covariance properties with a diffusion-based technique. This provided locally adapted smoothing to the representation with the application of a conductance function that locally controls the amount of diffusion, thus allowing the adaptation of the kernel’s width with regard to the analysis scale. Depending on the area being processed, the technique was able to discriminate high-value structured signal terms from lower-value weakly structured noise or likely interference term signals (and thus could identify and protect the structured components while smoothing the weakly structured ones, or rather, the interference terms). Thus, the readability of the representations was improved while the interference terms were mostly removed.

Gosme and Richard [40] proposed adaptive diffusion processes that locally controlled the amount of smoothing and the orientation of the applied smoothing, while enhancing localization and preserving covariance. This approach provided a framework not only for the Cohen class but also for the affine class. Variable conductance, general scheme, local smoothing strength, and orientation of the diffusion were suggested both for the Cohen and affine classes. A selective forward-and-backward diffusion process was investigated to prove its ability to remove cross-terms while reaching a high concentration for the signal components.

Gang and Xiao-niu introduced the pseudo-affine spectral correlation analysis, an alternative set of spectral correlation and class kernels [52]. The newly proposed pseudo-affine spectral correlation functions overcame issues of interference terms; the flexibility of the wavelet-based structure allowed continuous smoothing transition from the affine to the wavelet spectral correlation functions. In addition, the proposed spectral correlation functions were efficient for online computation and had the same resource requirements as the continuous wavelet transform. They suppressed interference terms by using a sliding structure that acted as proportional bandwidth smoothing. The introduced generators simplified the kernel formulations and helped the design of new affine distributions.

Gonçalvès et al. discussed approaches for constructing the affine class and the tools associated with it [17]. The advantages and shortcomings were discussed, different covariant classes were compared, and their interference terms and kernels were elaborated. In addition, new classes of time–frequency covariant distributions were introduced.

Gavrovska et al., in [53], proposed an algorithm for the detection of fundamental heart sounds S1 and S2 from phonocardiograms without the use of an ECG as a reference signal. The algorithm was based on a joint TFR from the pseudo-affine Wigner–Ville distribution, the Haar wavelet lifting scheme, the normalized average Shannon energy, and autocorrelation. The results were obtained using the algorithm on real-life healthy and pathological pediatric phonocardiogram signals, achieving a relatively high success rate without using a reference ECG signal.

Finally, Berge et al. [16] examined the Wigner distribution through a quantization perspective emphasizing the group structure. One of the main results was to express the scalogram as a convolution of affine distributions. In addition, the literature on affine Wigner distributions was reviewed, and a connection was made to the Mellin transform, with the affine ambiguity function presented; several applications were given.

Next, we provide a numerical analysis of the above-described TFRs in terms of their *t*–*f* concentration and IF estimation.

## 3. Examples and Simulation Results

Here we present simulation results and a comparison of three classes of TFRs: Cohen’s (classic), affine, and reassigned. The simulated examples were implemented in MATLAB with the help of the TFTB toolbox [13]. We also point interested readers to other freely available time–frequency signal analysis and processing toolboxes, such as the TFSAP [54,55] and the LTFAT [56]. The methods were tested on mono-component and multi-component synthetic signals (having constant, linear, parabolic, and sinusoidal FM) with additive noise, as well as on real-world signals. The evaluation Was performed in terms of IF estimation accuracy and TFR concentration. The estimated IF was calculated as in [10,11,12,57]:

(34) $$f_{e}\left(t\right)=arg\left\{\max_{f}\mathrm{TFR}\left(t,f\right)\right\},$$

where $f_{e}\left(t\right)$ is the estimated IF. Estimation of the IF from TFR maxima cannot be applied directly in the case of multi-component signals with components being present instantaneously; hence, component extraction was the step performed preceding IF estimation.

The analyzed TFRs from Cohen’s class include the spectrogram (SP), smoothed pseudo-Wigner–Ville (SPWV), and Wigner–Ville (WV) distributions. The tested affine class TFRs include affine Morlet wavelet scalogram (AMWSC), affine smoothed pseudo-Wigner–Ville (ASPWV), affine unitary Bertrand (AUB), affine active Unterberger (AAU), and affine D-Flandrin (ADF) representations, while from the reassigned class we considered the reassigned spectrogram (RSP), reassigned Gabor spectrogram (RGSP), reassigned Morlet scalogram (RMSC), reassigned pseudo-Wigner–Ville (RPWV), and reassigned smoothed pseudo-Wigner–Ville (RSPWV). The IF accuracy was assessed in terms of mean squared error (MSE), and the Rényi entropy was calculated for each TFR to evaluate the energy distribution concentration [58] and signal complexity [57] in the time–frequency domain as:

(35) $$R_{x}^{\alpha }=\frac{1}{1-\alpha }\log_{2}(\int_{-\infty }^{\infty }\int_{-\infty }^{\infty }{\mathrm{TFR}}_{x}^{\alpha }\left(t,f\right)dtdf),$$

where $R_{x}^{\alpha }$ is the α order Rényi entropy for the normalized ${\mathrm{TFR}}_{x}$ (in order to annul interferences contributions, we have set the Rényi entropy order to α=3, as in [22,59]).

### 3.1. Examples of Synthetic Signals

The methods from the three considered classes of TFRs were tested on synthetic, noisy (corrupted by additive white Gaussian noise with SNR ranging from 10 dB to −5 dB) signals having constant FM, linear FM (chirp), parabolic FM, and sinusoidal FM. The TFR plots for noisy signals are shown for a single noise realization, while the reported tables contain the MSE values and the Rényi entropies averaged over 100 iterations of random noise applied to the signal.

#### 3.1.1. Examples of Mono-Component Noisy Signals

First, we compare the Cohen’s, affine, and reassigned classes of TFRs in the time–frequency domain for a mono-component noisy chirp signal.

**Figure 3:**
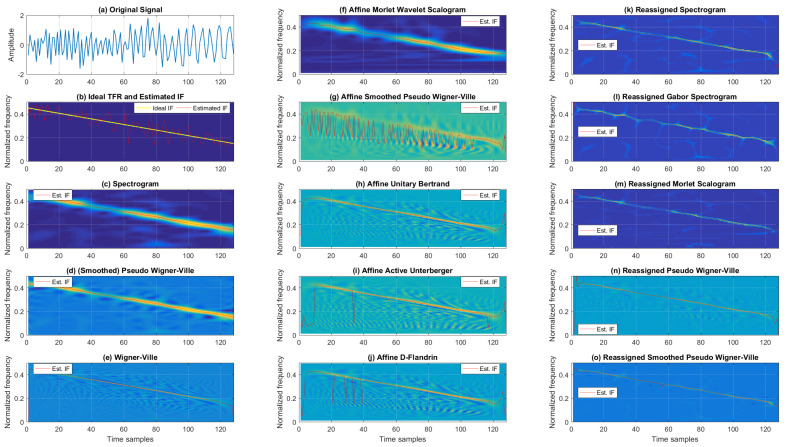
TFRs of a noisy mono-component linear FM signal with estimated IFs for the SNR of 5 dB.

Figure 3 shows the TFRs and IFs for the noisy chirp signal for 5 dB SNR. When considering classic TFRs, as expected, the best resolution was achieved by WV. On the other hand, the TFR from Cohen’s class that provided a balance between cross-terms and TF resolutions was SPWV. At the same time, SP resulted in the poorest TF resolution and reduced cross-terms. Next, the affine class TFRs are given in the second column of Figure 3, where AMWSC offered similar performance to the SP, achieving poorer resolution with significantly reduced cross-terms. Visual inspection shows a similar performance for AUB, AAU, and ADF, with numerous cross-terms and relatively good resolution. ASPWV offered an acceptable trade-off between cross-terms and resolution compared to the aforementioned affine TFRs for the noisy chirp signal. The reassigned TFRs are listed in the third column of Figure 3. RSP, RGSP, and RMSC acted rather similarly, exhibiting slightly poorer resolution with different levels of noise (while RPWV and RSPWV presented better resolution with residual cross-terms, with the latter performing significantly better with regard to cross-terms).

The IF estimation results, in terms of the MSE, for the noisy chirp signal are provided in Table 1. In general, as shown in the provided numerical results, Cohen’s class offered the most accurate IF estimation for the noisy, linear FM signal (with WV performing the best for intensive noise of −5 dB SNR, and SPWV performing the best for other tested SNRs). On the other hand, the worst performance for this type of signal for all SNRs was the affine class (to be more specific, ASPWV, except for the noise case of −5 dB SNR where AAU yielded the highest IF estimation MSE). From the reassigned class, RSPWV was the best for all SNRs, except for −5 dB, where it was outperformed by RPWV. The classic and reassigned TFRs exhibited better performance in comparison with affine TFRs for the tested signal.

In addition to the IF estimation accuracy, interesting conclusions can be drawn from Table 2 showing the Rényi entropy values for each tested TFR of the noisy chirp signal. Here we find that TFRs belonging to Cohen’s class resulted in the highest Rényi entropy values compared to the affine and reassigned classes for all tested SNRs. The best-performing TFRs, in terms of distribution concentrations, from the reassigned class were RSPWV for higher SNRs (10 dB and 5 dB) and RMSC for lower SNRs (0 dB and −5 dB). Interestingly, RSPWV reduced the Rényi entropy by 19.61% and 17.60% compared to the worst-performing TFRs from Cohen’s class, respectively (SP for 10 dB, WV for 5 dB). On the other hand, RMSC reduced the Rényi entropy by 17.09% and 15.43% compared to the worst-performing WV for 0 dB and −5 dB, respectively. As another example of a mono-component signal, let us consider a noisy, sinusoidal FM signal.

**Figure 4:**
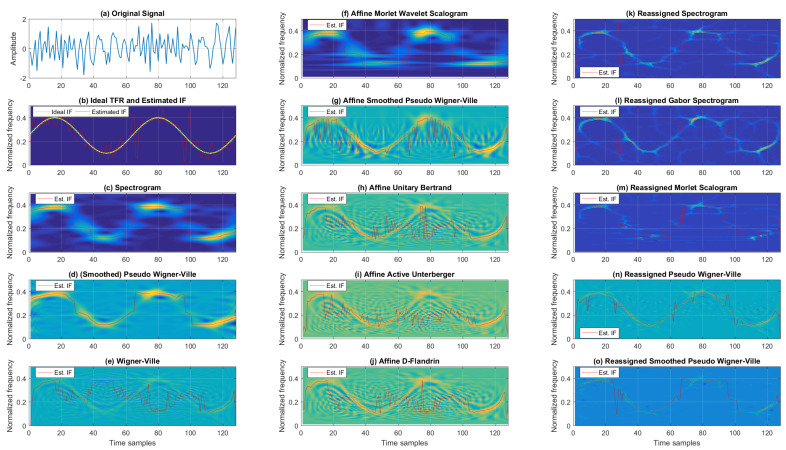
TFRs of a noisy mono-component sinusoidal FM signal with estimated IFs for the SNR of 5 dB.

TFRs from the Cohen’s, affine, and reassigned classes for the mono-component, noisy, sinusoidal FM signal are found in Figure 4. Here, when considering the classic TFR class, the WV showed severe cross-terms and better resolution than SP and SPWV (SPWV outperformed SP in terms of resolution, having cross-terms suppressed). Next, considering the affine TFRs, ADF, AAU, and AUB offered comparable performance, with relatively poor resolution and partly reduced cross-terms. AMWSC visually performed similarly to SP, with less resolution in the low-frequency range. ASPWV offered reduced cross-terms with rather reasonable resolution. Lastly, from the reassigned TFRs, RGSP, RSP, and RMSC had slightly poorer resolutions (the last being the worst-performing).

On the other hand, the RPWV and RSPWV had better resolution, with the first having faint cross-terms.

A quantitative comparison of the analyzed TFRs for this example is given in Table 3, which presents the estimated IF MSE from each tested TFR for a noisy, mono-component sinusoidal FM signal. Unlike in the case of the linear FM signal, here we find a significant difference between low and intensive noise. Namely, for low noise, the reassigned class significantly outperformed Cohen’s class, decreasing the MSE from $3.09\;\times \;10^{-4}$ and $6.05\;\times \;10^{-4}$ (achieved for the best-performing SPWV from Cohen’s class) to $1.06\;\times \;10^{-4}$ and $8.08\;\times \;10^{-4}$ (achieved for the best-performing RGSP and RSPWV from the reassigned class) for SNRs of 10 dB and 5 dB, respectively. The affine class performed poorly, with only WV providing a higher IF estimation MSE. On the other hand, in the case of intensive noise (0 dB and −5 dB SNR), SP and SPWV from Cohen’s class performed better than the reassigned and affine class TFRs (except for RPWV for −5 dB SNR, which decreased MSE in comparison to SPWV from $1.51\;\times \;10^{-2}$ to $1.46\;\times \;10^{-2}$). Again, the affine class performed poorly, with AAU resulting in the largest MSE.

The consistency in terms of TFR entropy-based concentration for different SNRs for this example can be observed in Table 4, which reports the Rényi entropy values for each tested TFR. Simulation results show the reassigned TFRs achieved the lowest Rényi entropy values (with negligible differences inside the class), and classic TFRs had the highest entropy values (with WV yielding the highest entropy for all SNRs). For the 10 dB, 5 dB, and 0 dB scenarios, the Rényi entropy ranged from 13.368, 13.587, and 13.923 (for RGSP) to 16.118, 16.355, and 16.569 (for WV). Finally, for −5 dB, the TFR entropy ranged from 14.207 for RMSC to 16.690 for WV. The affine TFRs demonstrated medium performance compared to the other two classes. On average, the best-performing TFRs for each class, in the case of the sinusoidal mono-component FM signal, were RGSP, AMWSC, and SPWV.

#### 3.1.2. Example of a Multi-Component Noisy Successive Signal

We continue our analysis for multi-component noisy synthetic signals. The first one to be analyzed is a signal consisting of multiple successive parabolic and linear FM components corrupted by additive white Gaussian noise.

**Figure 5:**
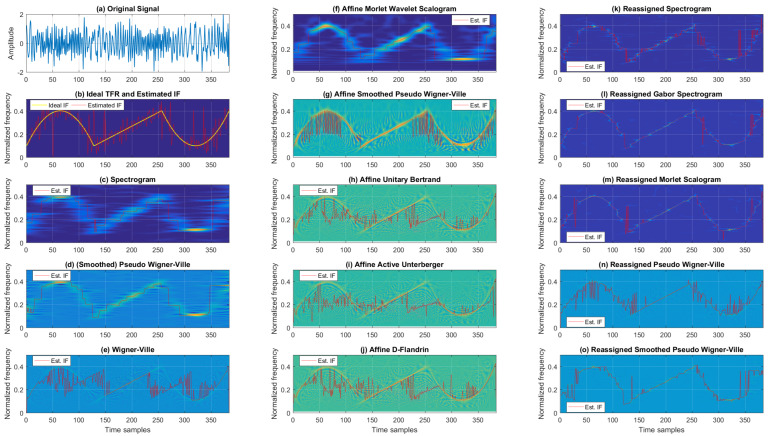
TFRs of a noisy multi-component signal with successive parabolic and linear FM components for 5 dB SNR.

Examples of TFRs of a noisy multi-component signal with successive parabolic and linear FM components for the SNR of 5 dB are given in Figure 5. Cohen’s class of TFRs (the first column in Figure 5) varied from good resolution and numerous cross-terms for WV to poor resolution with reduced cross-terms for SP, while SPWV provided a reasonable trade-off between the two. Next, the second column of Figure 5 presents the TFRs from the affine class, with AMWSC having similar performance to SP with visually somewhat better resolution (with a loss of resolution for the lower frequencies). AUB, AAU, and ADF visually performed similarly, with relatively good resolution, but they were also affected by cross-terms. ASPWV had moderately poorer resolution with a decrease in cross-term content. Lastly, from the reassigned TFRs, RSP, RGSP, and RMSC visually performed similarly (having acceptable resolution). RPWV and RSPWV presented good resolution, but both suffered from cross-terms.

MSEs of the estimated IF of a noisy multi-component signal with successive parabolic and linear FM components are given in Table 5. In the case of higher SNRs (10 dB and 5 dB) the IF MSE for Cohen’s class was minimal for SPWV (67.96 × 10^−5^ and 136.99 × 10^−5^, respectively). From the affine class, AMWSC outperformed other TFRs achieving 76.27 × 10^−5^ and 113.90 × 10^−5^ for SNRs of 10 dB and 5 dB, respectively. From the reassigned class, for SNRs of 10 dB and 5 dB, RGSP outperformed other reassigned TFRs, achieving MSEs of 9.39 × 10^−5^ and 39.73 × 10^−5^, respectively. When considering intensive noise scenarios, it is interesting to note that SP offers the most accurate IF estimation for 0 dB (304.51 × 10^−5^). For comparison, the worst-performing was AAU from the affine class, resulting in an IF estimation MSE of 1774.30 × 10^−5^. Similar conclusions can be drawn for the case of an SNR of −5 dB, where SP’s IF estimation MSE was 1125.70 × 10^−5^ (negligibly outperformed by RPWV with an MSE of 1082.20 × 10^−5^). Again, as for 0 dB, AAU was the worst-performing with 2321.90 × 10^−5^. Thus, Cohen’s class resulted in the most accurate IF estimation for intensive noise SNRs for the noisy multi-component signal with successive parabolic and linear FM components, while the reassigned class of TFRs was the best-performing for low-noise environments. In both cases, the affine class was outperformed by the other two classes.

Unlike in the case of IF estimation, where the reassigned class was the best choice for low-noise and Cohen’s class was best for high-noise scenarios, when considering the Rényi entropy of TFRs of a signal with successive parabolic and linear FM components, the best-performing class for all tested SNRs was the reassigned class. This is evident from Table 6, where it can also be seen that Cohen’s class performs, in general, worse than the affine class for this type of signal in terms of TFR concentration. The worst-performing of all tested SNRs was WV, while the worst-performing affine class TFR, AAU, outperformed WV by up to 2.76%, 1.99%, 1.27%, and 0.77% for SNRs 10, 5, 0, and −5 dB, respectively. On the other hand, the best-performing reassigned TFR for all tested SNRs was RMSC, which reduced the Rényi entropy by 19.86%, 18.77%, 16.55%, and 14.42% for SNRs 10, 5, 0, and −5 dB, respectively, compared to WV.

#### 3.1.3. Example of a Multi-Component Noisy Concurrent Signal

**Figure 6:**
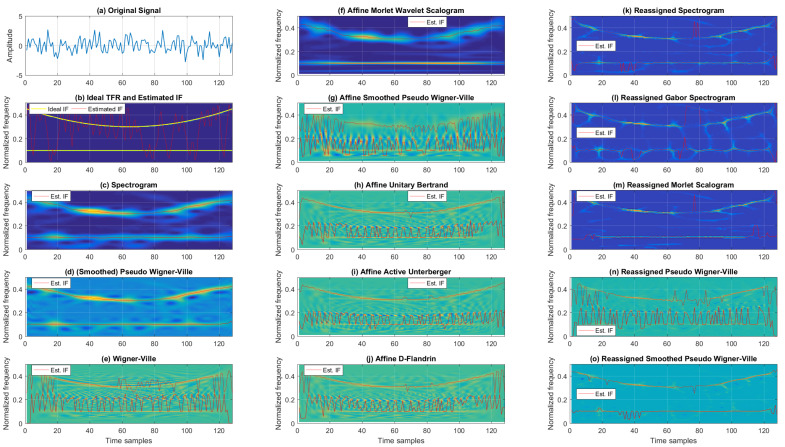
TFRs of a noisy multi-component signal with concurrent constant and parabolic FM components for the 5 dB SNR.

We conclude the simulation analysis of synthetic signals with another example of a multi-component noisy signal consisting of concurrent constant and parabolic FM components with additive Gaussian noise. For visual assessment, in Figure 6, we present TFRs from the Cohen’s, affine, and reassigned classes for 5 dB SNR. Again, from the classic TFRs, SP had poor resolution, SPWV achieved better resolution with some cross-terms, and WV had even better resolution (however, with severe interference terms). From the affine class, AUB, AAU, and ADF visually had similar performance (balancing time–frequency resolution and amount of cross-terms). ASPWV had poorer resolution; however, it also had weaker cross-terms. AMWSC displayed a similar performance to the scalogram. Lastly, from the reassigned TFRs, RSP, RGSP, and RMSC visually performed similarly with somewhat poorer resolution.

RPWV and RSPWV presented satisfactory resolution, with the former exhibiting more accentuated cross-terms.

MSE for the estimated IF for the noisy multi-component signal with concurrent constant and parabolic FM components is given in Table 7. It can be seen that the affine class AMWSC provided, in general, the lowest MSE for all tested SNRs. For the 10 dB SNR, AMWSC reduced the MSE by 75.48 % and 29.84% compared to the second-best (SP) for the first and second components, respectively. For 5 dB, AMWSC reduced MSE by 90.41% compared to the second-best (SP) for the first component, while for the second component, SP performed somewhat better, resulting in an MSE of $8.04\;\times \;10^{-6}$ (AMWSC obtained $8.81\;\times \;10^{-6}$). For 0 dB, AMWSC performed well, with MSE being reduced by 93.19% for the first component compared to the second-best (SPWV), and MSE increased by 5.01% compared to SPWV for the second component. Moreover, for an intensive noise environment (−5 dB SNR), AMWSC reduced the MSE by 61.68% and 0.32% (for the first and second components, respectively) compared to the second-best from the affine class (AAU).

TFR Rényi entropy results for a noisy multi-component signal with concurrent constant and parabolic FM components for different SNR levels (averaged over 100 noise realizations) is presented in Table 8. The Rényi entropy ranged from 13.308 to 16.698, with those values belonging to RSPWV for the SNR of 10 dB and WV for an SNR of −5 dB, respectively. The results show that the reassigned TFRs outperformed those from the Cohen’s and affine classes by up to 18.01%, 16.28%, 15.34%, and 14.33% for 10 dB, 5 dB, 0 dB, and −5 dB SNRs, respectively (with Cohen’s class WV resulting in the highest Rényi entropy for all SNRs). In terms of the entropy-based distribution concentration for this multi-component signal, the best-performing TFRs on average per class were AMWSC for the affine class, SPWV for Cohen’s class, and RSPWV and RMSC from the reassigned class for low and intense noise, respectively.

### 3.2. Examples of Real-World Signals

Next, we present the performance of the Cohen’s, affine, and reassigned class distributions using two real-world examples, in terms of concentration, measured by the Rényi entropy.

First, we analyzed the concentrations of TFRs for an electroencephalogram (EEG) signal, observing the P300 response to external stimuli. The multichannel dataset consisted of 32 channels measuring brain activity and 942 trials with and without external stimuli [60]. We focused on the Cz electrode and analyzed the signal obtained from the average of 466 responses to stimuli.

**Figure 7:**
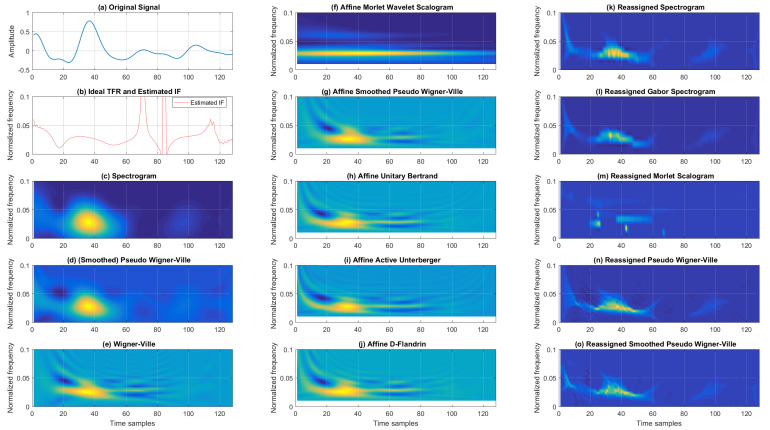
TFRs of an EEG P300 signal for the Cz electrode (averaged over 466 trials).

Figure 7 gives the time–frequency distributions of the analyzed EEG P300 signal. A simple visual inspection of the classic group of TFRs shows that SP had poor concentration, which was improved on by SPWV at the cost of interference terms. WV showed even better resolution, but was more deteriorated by cross-terms. From the affine TFRs, the poorest concentration was found for AMWSC, followed by AUB, AAU, and ADF. ASPWV had moderately poorer concentration; however, it had fewer cross-terms. From the reassigned TFRs, RSP and RGSP achieved similar performance. RMSC performed with acceptable concentration, and RPWV and RSPWV visually performed similarly, showing satisfactory concentration, with the latter performing better regarding cross-terms.

A numerical comparison of the analyzed TFRs’ for the EEG P300 signal is given in Table 9. The Rényi entropy ranges between 10.178 and 16.689. The classic TFRs exhibited average performance, the affine TFRs had poorer performance, and the reassigned TFRs achieved the best performance. The best- and worst-performing TFRs, when considering all TFR classes, were RMSC and AMWSC. The best reassigned TFR (RMSC) outperformed the worst TFR (affine AMWSC) by 39.01%, and the best TFR from the Cohen class (SPWV), by 20.6%.

Finally, we provide the comparison of the analyzed TFRs on another real-world example: the vertical acceleration seismogram of the earthquake that hit Zagreb, the capital of Croatia, on 22 March 2020. The magnitude was 5.5 according to the Richter scale, making it the strongest earthquake to hit Zagreb since the 1880s. The depth at the epicenter was 10 km, and the epicenter was 7 km north of the city center. The closest surveying station was located in the city and municipality of Lobor, some 38 km from the capital of Zagreb, where the seismogram signal was recorded.

**Figure 8:**
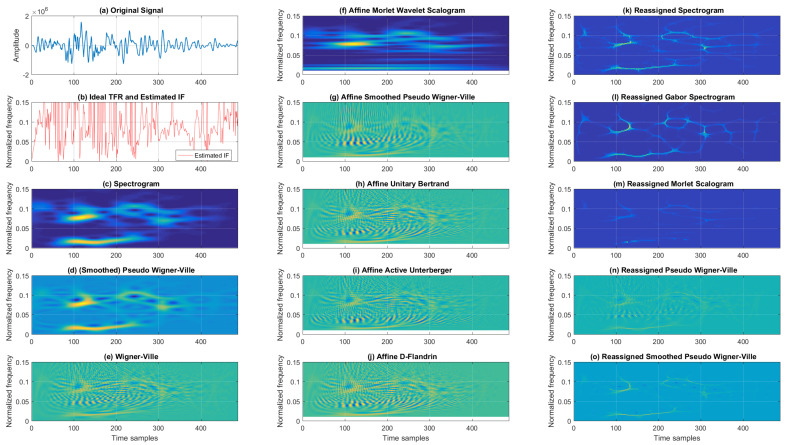
TFRs of the 2020 Zagreb earthquake vertical acceleration seismogram.

TFRs of the earthquake signals are shown in Figure 8. As in the previous real-life example, for Cohen’s class, WV achieved the best resolution and most cross-terms, as opposed to SP for both features. SPWV, as already seen, provided a trade-off between resolution and cross-terms. When considering the affine group of TFRs, AMWSC presented poor concentration according to visual inspection, while AUB, AAU, and ADF had intense cross-terms compared to ASPWV. From the reassigned TFRs, RSP and RGSP showed relatively good concentration, which was further improved for RMSC. RPWV visually showed good concentration, but it was also affected by cross-terms, whilst RSPWV showed acceptable performance with regard to concentration, with the presence of few cross-terms.

Table 10 shows the TFR Rényi entropies for the Zagreb 2020 earthquake vertical acceleration seismogram signal. The Rényi entropy values ranged from a minimum of 15.084 to a maximum of 21.526. It can be seen that the TFRs assume class-related behavior, with the affine group performing the worst, the classic group of TFRs performing in the middle, and the reassigned group of TFRs being the best in terms of the Rényi entropy concentration. The best-performing TFRs per group were SPWV for the classic group, ASPWV for the affine class, and RMSC for the reassigned class. The best-performing TFR for this example, in general, was RMSC, and the worst-performing was AMWSC. The best reassigned TFR (RMSC) outperformed the worst TFR (affine AMWSC) by 29.92%, and the best TFR from the Cohen class (SPWV) by 12.33%, in the case of the tested earthquake signal.

### 3.3. Computational Cost of Analyzed TFRs

Here we present the computational cost of the tested methods. The TFRs were computed in MATLAB (R2016a version) on Windows 10. As a computational platform, we used an HP Pavilion Power 17-ab307nm 2ZJ05EA with an Intel^®^ Core™ i5-7300HQ CPU @ 2.50 GHz, 16 GB of DDR4-2666 (1333 MHz) RAM, and NVIDIA GeForce GTX 1050 Ti GPU with 4 GB GDDR5 of dedicated memory (7.9 GB total shared memory).

Table 11 shows the computational cost of calculating each of the analyzed TFRs from previous examples (averaged over 100 calculations for an SNR of 5 dB). The fastest TFRs, in terms of computational CPU time, were WV, SP, RPWV, and AMWSC, while the computationally most demanding was AAU. As for groups, Cohen’s class performed the fastest, followed by the reassigned class and then the affine class. In addition, as expected, signal length significantly affected the computational cost. To study the effect of signal length on computational cost in more depth, we present CPU computational times in Table 12 for an example of a linear FM signal with a different number of signal samples (averaged over 100 calculations).

Next, in terms of the computational cost of analyzed TFDs, we present the computational time for calculating TFRs of different dimensions, as shown in Table 13. As shown, in general, a larger TFR dimension led to slower TFR calculations, especially for the affine and reassigned classes, while for Cohen’s class, this increase in CPU computational time was not so significant.

In conclusion, this paper provides novel insight into the performances of TFRs from the Cohen’s, affine, and reassigned classes in terms of their IF estimation, entropy-based concentration, and computational cost. To the best of our knowledge, based on an extensive literature review, there exists no similar study comparing SP, WV, SPWV, AMWSC, ASPWV, AUB, AAU, ADF, RSP, RGSP, RMSC, RPWV, and RSPWV on both synthetic (mono-component and multi-component) signals in noise for different SNR levels, as well as on the real-time examples. The conclusions and findings of this research may help in selecting the appropriate TFR for a signal of interest to be analyzed.

As a direction for future work, we suggest designing new adaptive TFRs optimized for IF estimation and/or distribution concentration and applying these in fields where classical TFRs are often used, with the expectation of outperforming them.

## 4. Results Discussion

This study compares the performance of three classes of TFRs (Cohen’s, affine, and reassigned) for different signals (mono-component and multi-component), different frequency modulations, and different noise levels. As it is visible from the reported results, there is no one particular TFR that performs best in all tested scenarios in terms of IF estimation accuracy and TF concentration.

In general, when considering Cohen’s class, consistently good performance was achieved by SPWV. From the affine class, MWSC and ASPWV performed relatively well, while RSPWV and RPWV should be considered as the first choices from the reassigned class.

When comparing TFR classes, the classic Cohen’s and reassigned TFRs outperformed the affine group with regard to the MSE of the estimated IF. In general, for most cases and SNRs, the Cohen’s class resulted in improved IF estimation, while the reassigned class resulted in somewhat higher MSE values. Finally, the affine class saw the least change in performance with the change in the SNR, having mostly medium to poor performance, with a few exceptions.

According to the Rényi entropy measure, the reassigned class performed best, followed by the affine and Cohen’s class TFRs, with some exceptions. The best-performing TFRs per group were AMWSC, SPWV, and RMSC. Over most of the examples and SNRs, the best-performing TFRs were RMSC, RSP, RSPWV, and RGSP.

The best-performing TFR of the reassigned class was RSPWV, followed by RMS and RGS (which was more sensitive to noise). The best-performing TFRs from Cohen’s class were SP (having relatively poor concentration) and SPWV (presenting some residual cross-terms). Finally, in the case of the affine class, visually, the best-performing was AMWSC, with a poorer concentration in the lower frequency bands, and ASPWV, which suffered from cross-terms.

According to the Rényi entropy, used as a measure of TFR concentration, the affine class of TFRs achieved poor performance, Cohen’s class TFRs displayed medium performance, while the reassigned class resulted in the best performance for the real-life examples. In general, the best TFR, according to the Rényi entropy, for tested real-world examples was RSPWV, followed by RMS, RGS, and RS. When compared at a class level, the best TFRs for analyzed real-life examples were ASPWV in the affine class, SPWV in Cohen’s class, and RMS in the reassigned class.

## 5. Conclusions

This paper elaborated on and compares three classes of TFRs: Cohen’s, affine, and reassigned, including the theoretical background of the selected TFRs belonging to these classes. Next, we performed extensive numerical simulations on non-stationary signals, including both synthetic and real-life examples. TFD quality was evaluated with respect to IF estimation accuracy, TF concentration measured by the Rényi entropy, and the presence of cross-terms.

As shown, the performances of the TFRs were highly affected by the type of signal being analyzed. When choosing the proper TFR, one should consider the following parameters: the presence and intensity of noise, the number of signal components, and the FM of signal components, to name a few.

The best-performing TFRs in terms of the IF estimation MSE, for the mono-component linear FM signal, were WV and SPWV, depending on the noise intensity. In the case of the mono-component sinusoidal FM signal, the best-performing TFRs were RGSP, RSPWV, SP, and SPWV as the noise intensified. On the other hand, the best for the successive multi-component signal was RGSP for lower noise and SP for high noise. For the concurrent multi-component signal, AMWSC outperformed all other TFRs.

Regarding the Rényi entropy, the best-performing TFRs for mono-component linear FM signals were RSPWV for lower noise and RMSC for higher noise. For the sinusoidal signal, the best TFRs were RGSP and RMSC, depending on the noise level. For the successive multi-component FM signal, RMSC outperformed other TFRS for all tested SNRs, while for the concurrent multi-component signal, the best TFRs were RSPWV for the higher SNRs and RMSC for the lower SNRs.

Furthermore, there is often a compromise between the time–frequency resolution and cross-term presence, as well as between the accuracy of the IF estimation and TF concentration.

Since there is no one particular TFR with superior performance for all cases, this study presents extensive insight into the behavior of the analyzed TFRs classes, providing help in choosing the appropriate representation for the analyzed signal of a non-stationary phenomenon.

In future work, we plan to focus on developing new adaptive TFRs (from the Cohen’s, affine, and reassigned classes) optimized for IF estimation and/or entropy-based concentration and to apply these adaptive TFRs in cases where classical TFRs are often utilized, with the expectation of improving upon their performance.

## Figures and Tables

**Table 1:** Estimation of IF MSE for noisy mono-component linear FM signal for different SNR levels (averaged over 100 noise realizations), cell colors range from green for the best result to red for the worst result in each column respectively, 1 × 10^−5^.

	10 dB	5 dB	0 dB	−5 dB
SP	1.58	2.39	111.24	1329.70
SPWV	1.46	2.08	98.58	1320.50
WV	1.64	34.19	231.40	918.89
AMWSC	4.61	9.40	326.76	2039.20
ASPWV	277.84	695.28	1789.10	2647.70
AUB	31.88	77.71	706.32	2093.80
AAU	75.61	280.15	1667.80	3137.30
ADF	66.84	181.90	1249.60	2381.20
RSP	11.88	35.94	497.45	1811.40
RGSP	13.09	37.51	683.30	2014.00
RMSC	22.85	88.66	609.31	1662.40
RPWV	42.16	100.22	521.22	1288.90
RSPWV	1.57	11.84	343.78	1645.20

**Table 2:** TFR Rényi entropy for a noisy mono-component linear FM signal for different SNR levels (averaged over 100 noise realizations), cell colors range from green for the best result to red for the worst result in each column respectively.

	10 dB	5 dB	0 dB	−5 dB
SP	15.225	15.475	15.959	16.480
SPWV	14.905	15.293	15.898	16.437
WV	14.864	15.600	16.343	16.641
AMWSC	13.774	14.242	15.078	15.883
ASPWV	15.022	15.551	15.933	16.134
AUB	14.193	15.071	15.875	16.232
AAU	14.690	15.516	16.199	16.446
ADF	14.301	15.191	15.919	16.187
RSP	12.886	13.172	13.682	14.187
RGSP	12.972	13.272	13.742	14.174
RMSC	12.796	13.061	13.550	14.074
RPWV	13.107	14.018	14.800	15.104
RSPWV	12.240	12.854	13.684	14.349

**Table 3:** Estimation of IF MSE for a noisy mono-component sinusoidal FM signal for different SNR levels (averaged over 100 noise realizations), cell colors range from green for the best result to red for the worst result in each column respectively, 1 × 10^−4^.

	10 dB	5 dB	0 dB	−5 dB
SP	6.88	10.28	36.54	153.62
SPWV	3.09	6.05	34.86	150.80
WV	187.12	153.31	135.68	150.92
AMWSC	17.83	26.08	73.08	197.99
ASPWV	11.58	60.63	150.89	217.60
AUB	92.30	97.53	134.96	185.98
AAU	99.05	127.31	187.59	245.58
ADF	100.06	109.18	152.09	200.02
RSP	1.79	8.54	72.52	197.80
RGSP	1.06	8.68	90.08	217.16
RMSC	7.93	24.19	89.60	197.35
RPWV	2.74	13.09	65.93	145.99
RSPWV	1.92	8.08	62.18	184.94

**Table 4:** TFR Rényi entropy for a noisy mono-component sinusoidal FM signal for different SNR levels (averaged over 100 noise realizations), cell colors range from green for the best result to red for the worst result in each column respectively.

	10 dB	5 dB	0 dB	−5 dB
SP	15.947	16.111	16.381	16.654
SPWV	15.724	15.973	16.300	16.586
WV	16.118	16.355	16.569	16.690
AMWSC	15.170	15.402	15.788	16.227
ASPWV	15.416	15.871	16.080	16.240
AUB	15.482	15.765	16.070	16.340
AAU	15.615	15.956	16.269	16.518
ADF	15.499	15.783	16.058	16.278
RSP	13.579	13.720	13.998	14.301
RGSP	13.368	13.587	13.923	14.249
RMSC	13.522	13.689	13.991	14.207
RPWV	14.127	14.578	14.957	15.152
RSPWV	13.650	13.890	14.226	14.488

**Table 5:** Estimation of IF MSE for a noisy multi-component signal with successive parabolic and linear FM components for different SNR levels (averaged over 100 noise realizations), cell colors range from green for the best result to red for the worst result in each column respectively, 1 × 10^−5^.

	10 dB	5 dB	0 dB	−5 dB
SP	127.12	166.02	304.51	1125.70
SPWV	67.96	136.99	325.23	1160.50
WV	1046.80	1068.60	1061.80	1206.60
AMWSC	76.27	113.90	350.10	1501.40
ASPWV	148.89	480.46	1292.90	1816.10
AUB	684.84	783.60	1122.60	1546.40
AAU	848.84	1098.20	1774.30	2321.90
ADF	750.37	910.03	1337.60	1707.00
RSP	22.18	65.28	456.63	1509.00
RGSP	9.39	39.73	445.73	1513.40
RMSC	9.84	45.78	524.60	1540.00
RPWV	61.37	179.57	514.64	1082.20
RSPWV	42.95	107.03	475.40	1487.00

**Table 6:** TFR Rényi entropy for a noisy multi-component signal with successive parabolic and linear FM components for different SNR levels (averaged over 100 noise realizations), cell colors range from green for the best result to red for the worst result in each column respectively.

	10 dB	5 dB	0 dB	−5 dB
SP	19.616	19.839	20.226	20.519
SPWV	19.379	19.696	20.148	20.449
WV	20.097	20.301	20.518	20.637
AMWSC	18.577	18.932	19.561	20.118
ASPWV	19.184	19.742	20.108	20.267
AUB	19.325	19.658	20.027	20.274
AAU	19.542	19.897	20.258	20.478
ADF	19.358	19.692	20.027	20.231
RSP	16.639	16.916	17.416	17.869
RGSP	16.288	16.642	17.217	17.733
RMSC	16.105	16.491	17.122	17.662
RPWV	17.575	18.256	18.858	19.097
RSPWV	16.837	17.244	17.753	18.032

**Table 7:** Estimation of IF MSE for a noisy multi-component signal with concurrent constant and parabolic FM components for different SNR levels (averaged over 100 noise realizations), cell colors range from green for the best result to red for the worst result in each column respectively.

	10 dB	5 dB	0 dB	−5 dB
(a) First Component, 1 × 10^−6^				
SP	5.26	22.41	1032.10	3914.40
SPWV	7.60	38.28	1017.90	3877.20
WV	4375.40	6032.50	8521.80	9397.40
AMWSC	1.29	2.15	69.36	1446.50
ASPWV	3384.30	3910.00	4724.80	5443.90
AUB	4108.60	4593.10	5160.00	5430.20
AAU	3774.00	3900.40	4066.40	3775.90
ADF	4872.90	5352.50	5718.60	5528.00
RSP	227.92	404.64	1942.20	4245.50
RGSP	168.09	487.04	2243.00	4406.40
RMSC	508.97	930.73	1785.50	4107.40
RPWV	3648.10	4975.40	6204.10	6903.10
RSPWV	45.29	193.54	1584.20	3961.30
(b) Second Component, 1 × 10^−5^				
SP	5.16	8.04	136.88	483.73
SPWV	5.76	8.80	133.50	467.15
WV	237.34	306.31	405.21	447.99
AMWSC	3.62	8.81	140.19	444.00
ASPWV	400.74	450.91	557.93	635.48
AUB	49.20	94.12	298.59	452.07
AAU	19.29	54.31	273.11	445.41
ADF	137.54	216.86	408.39	508.84
RSP	26.17	52.88	288.19	624.45
RGSP	26.73	73.70	371.42	700.51
RMSC	20.90	39.49	277.02	600.25
RPWV	183.87	251.13	398.47	508.63
RSPWV	5.23	21.09	222.81	544.92

**Table 8:** TFR Rényi entropy for a noisy multi-component signal with concurrent linear and parabolic FM components for different SNR levels (averaged over 100 noise realizations), cell colors range from green for the best result to red for the worst result in each column respectively.

	10 dB	5 dB	0 dB	−5 dB
SP	16.033	16.168	16.392	16.643
SPWV	15.794	16.051	16.345	16.598
WV	16.232	16.453	16.625	16.698
AMWSC	14.642	14.946	15.497	16.125
ASPWV	15.738	16.030	16.182	16.271
AUB	15.239	15.710	16.122	16.342
AAU	15.260	15.780	16.233	16.483
ADF	15.287	15.737	16.099	16.277
RSP	13.820	13.971	14.162	14.372
RGSP	13.913	14.038	14.182	14.315
RMSC	13.615	13.786	14.075	14.305
RPWV	14.245	14.741	15.063	15.181
RSPWV	13.308	13.774	14.223	14.506

**Table 9:** TFR Rényi entropy of an EEG P300 signal for the Cz electrode (averaged over 466 trials), cell colors range from green for the best result to red for the worst result in each column respectively.

	Rényi Entropy
SP	13.414
SPWV	13.251
WV	14.366
AMWSC	16.689
ASPWV	16.200
AUB	16.300
AAU	16.302
ADF	16.325
RSP	11.369
RGSP	11.230
RMSC	10.178
RPWV	11.782
RSPWV	11.439

**Table 10:** Rényi entropy for the 2020 Zagreb earthquake vertical acceleration seismogram, cell colors range from green for the best result to red for the worst result in each column respectively.

	Rényi Entropy
SP	18.961
SPWV	18.871
WV	19.831
AMWSC	21.526
ASPWV	20.956
AUB	21.350
AAU	21.417
ADF	21.260
RSP	16.636
RGSP	16.441
RMSC	15.084
RPWV	18.099
RSPWV	16.494

**Table 11:** Computation time for analyzed TFRs for tested signals (averaged over 100 realizations, SNR of 5 dB), cell colors range from green for the best result to red for the worst result in each column respectively, in milliseconds (ms).

	Noisy Mono Component Linear FM	Noisy Mono Component Sinusoidal FM	Noisy Three Component Parabolic and Linear FM	Noisy Two Component Constant and Parabolic FM	EEG P300 Signal	2020 Zagreb Earthquake
Number of time samples	128	128	384	128	128	487
SP	1.6448	1.4987	7.4916	1.4124	1.4105	12.69
SPWV	23.024	22.617	225.12	22.428	22.569	369.87
WV	1.4677	1.3349	7.7009	1.2996	1.2905	13.539
AMWSC	10.219	9.7795	60.675	9.7801	9.6643	93.012
ASPWV	188.92	188.19	3122.5	188.94	189.51	4356.7
AUB	284.9	283.59	2110.8	281.91	283.34	2476.9
AAU	749.92	751.21	6348.1	745.8	748.24	7999
ADF	250.64	250.61	1797.7	250.28	251.04	2049.4
RSP	19.526	19.205	170.08	19.449	19.203	275.86
RGSP	16.126	15.649	145.11	15.565	13.163	192.5
RMSC	275.13	268.87	2650.7	273.55	269.98	4207.1
RPWV	3.7773	3.4253	24.559	3.4197	3.4948	41.555
RSPWV	44.623	43.586	412.51	43.931	43.878	670.22

**Table 12:** Computational time for analyzed TFRs for a noisy linear FM signal with different numbers of time samples (averaged over 100 realizations, SNR of 5 dB), cell colors range from green for the best result to red for the worst result in each column respectively, in milliseconds (ms).

Number of Time Samples	32	64	128	256	512
SP	0.53308	0.68926	1.4145	3.6583	10.057
SPWV	1.9574	5.7625	22.275	92.194	407.77
WV	0.38185	0.47453	1.2714	2.968	11.174
AMWSC	2.2736	3.9472	9.6087	28.944	104.7
ASPWV	26.755	47.652	179.4	455.84	4537.6
AUB	46.272	103.14	278.18	791.83	2500.5
AAU	99.284	257.56	727.06	2276.1	8031.1
ADF	42.68	94.99	244.54	681.51	2116.2
RSP	2.2171	5.4668	18.87	70.807	285.32
RGSP	2.1111	5.0283	15.18	51.456	201.96
RMSC	16.188	63.321	261.1	1110.3	4600.3
RPWV	0.99658	1.3783	3.3517	9.9509	38.288
RSPWV	3.8662	11.514	42.46	169.57	721.94

**Table 13:** Computational time for analyzed TFRs of different dimensions for a noisy linear FM signal with 128 time samples (averaged over 100 realizations, SNR of 5 dB), cell colors range from green for the best result to red for the worst result in each column respectively, in milliseconds (ms).

TFR Dimension	32×32	64×64	128×128	256×256	512×512
SP	1.4193	1.3427	1.4466	1.6453	2.0954
SPWV	23.058	22.478	22.415	23.576	24.098
WV	1.0921	1.0986	1.2774	1.3618	1.5274
AMWSC	3.1886	5.0576	9.607	19.048	37.809
ASPWV	34.879	57.728	101.24	188.75	447.8
AUB	19.101	28.757	48.061	88.604	160.95
AAU	26.891	41.811	73.08	126.18	228.62
ADF	16.001	25.113	43.079	75.009	141.32
RSP	7.2236	11.604	19.851	38.007	72.442
RGSP	5.8219	9.1319	16.085	29.338	57.913
RMSC	66.308	134.42	269.46	542.5	1129.2
RPWV	2.8339	2.7727	3.3549	4.8437	8.2814
RSPWV	30.529	34.782	42.888	59.86	95.77

## Data Availability

Not applicable.

## Abbreviations

The following abbreviations are used in this manuscript:

TFR	Time–Frequency Representation
TFD	Time–Frequency Distribution
TSR	Time–Scale Representation
TSD	Time–Scale Distribution
IF	Instantaneous Frequency
TF	Time–Frequency
FM	Frequency Modulation
SNR	Signal-to-Noise Ratio
MSE	Mean Squared Error
SP	Spectrogram
SPAWD	Smoothed Pseudo-Affine Wigner Distribution
SPWV	Smoothed Pseudo-Wigner–Ville
WV	Wigner–Ville
AMWSC	Affine Morlet Wavelet Scalogram
ASPWV	Affine Smoothed Pseudo-Wigner–Ville
AUB	Affine Unitary Bertrand
AUU	Affine Active Unterberger
ADF	Affine D-Flandrin
RSP	Reassigned Spectrogram
RGSP	Reassigned Gabor Spectrogram
RMSC	Reassigned Morlet Scalogram
RPWV	Reassigned Pseudo-Wigner–Ville
RSPWV	Reassigned Smoothed Pseudo-Wigner–Ville
